# Significance of Oral Manifestations in the Diagnosis of Severe Phenytoin-Induced Thrombocytopenia: A Rare Case

**DOI:** 10.7759/cureus.37585

**Published:** 2023-04-14

**Authors:** Vinodh Gangadaran, Manonmani Balasubramanian

**Affiliations:** 1 Department of Dentistry, Kilpauk Medical College and Hospital, Chennai, IND; 2 Department of Oral and Maxillofacial Surgery, Thai Moogambigai Dental College and Hospital, Chennai, IND

**Keywords:** hemorrhage, phenytoin, thrombocytopenia, hemorrhagic, oral manifestation, early diagnosis

## Abstract

Phenytoin is a commonly used anticonvulsant drug for the prophylaxis of generalized tonic-clonic seizures, partial seizures, and neurosurgery-related seizure prevention. Phenytoin-induced thrombocytopenia is a rare but life-threatening condition. Close monitoring of blood counts may be necessary for patients receiving phenytoin, as delay in diagnosis or withdrawal of the drug can be life-threatening. Clinical manifestations of phenytoin-induced thrombocytopenia are usually reported within one to three weeks after drug initiation. In this article, we report a unique case of drug-induced thrombocytopenia that manifested as multiple hemorrhagic lesions in the oral mucous membrane three months after phenytoin initiation.

## Introduction

Drug-induced thrombocytopenia (DIT) has been reported by many authors. DIT was first reported by Vipan in 1865 in patients treated with quinine, with clinical manifestations of purpura [1]. A platelet count of less than 150 x 10^9^/L is defined as thrombocytopenia. Clinical manifestations of thrombocytopenia include petechiae, purpura, and hemorrhage in cases of severe thrombocytopenia. Phenytoin has been used for the treatment of seizures since 1938. German professor Heinrich Biltz synthesized phenytoin (5,5-Diphenylhydantoin) in 1908 [2]. In 1938, Tracy Putnam and H. Houston Merritt discovered its use in seizure control [3].

Pedersen-Bjergaard et al.'s [4] report showed that the incidence rate of DIT with anticonvulsants was 0.96 in 100,000 prescriptions in a year. Blackburn et al.'s cohort study showed that the incidence rate of DIT varied among each class of anticonvulsant drugs [5]. The incidence rate with carbamazepine was 0.5 in 100,000 prescriptions, 1.1 in 100,000 prescriptions of phenytoin, 1.6 in 100,000 prescriptions of valproate, and 4.2 in 100,000 prescriptions of phenobarbital [5]. Thorning et al. reported a fatal case of phenytoin-induced thrombocytopenia in a neurosurgical patient in 2007.

Phenytoin-induced thrombocytopenia usually occurs within one to three weeks after exposure to the drug, but in some cases, the time of onset may vary from two days to two years after initiation of phenytoin treatment [6]. We report a case of phenytoin-induced thrombocytopenia that presented with multiple oral hemorrhagic manifestations and one purpuric spot in the left upper arm three months after initiation of phenytoin.

## Case presentation

A 45-year-old female patient presented with complaints of occasional bleeding from her gums for the past five days and severe bleeding from her gums for one day. She had a history of seizures following a road traffic accident three months ago, and a CT scan taken at the time of the trauma showed evidence of intracranial bleeding, for which the patient was prescribed phenytoin 100 mg three times a day as prophylaxis. There were no signs of epistaxis, hematuria, melena, or menorrhagia and no history of predisposing comorbid conditions like hypertension, anticoagulation therapy, or recent surgery.

**Figure 1:**
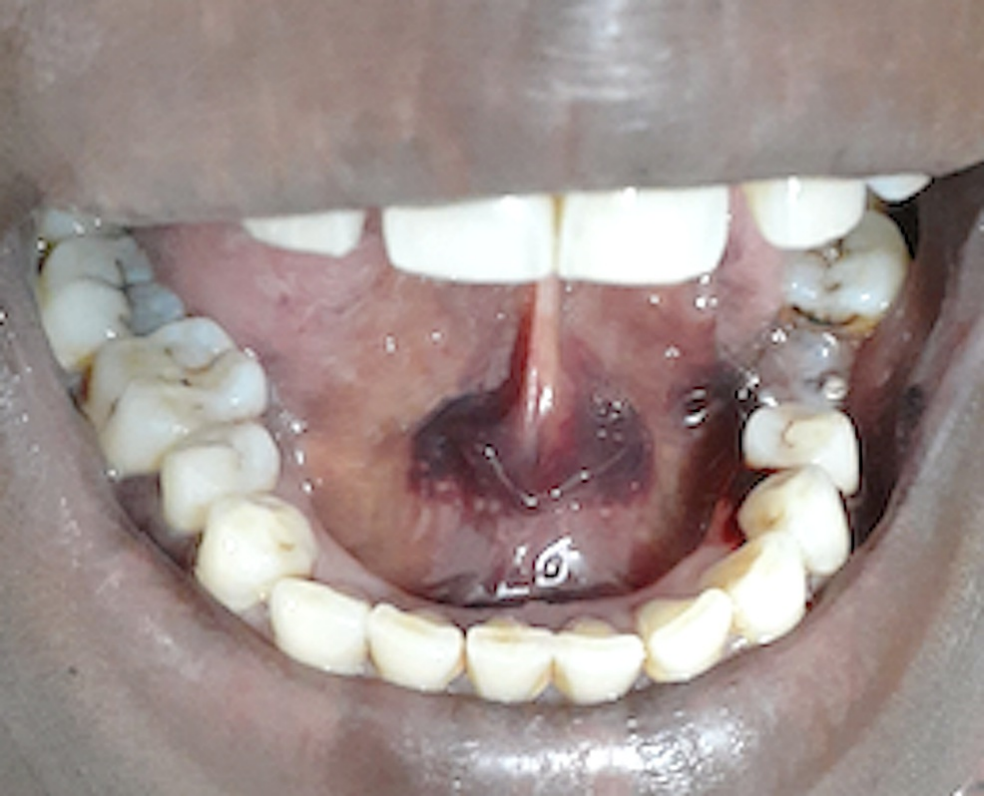
Phenytoin-induced thrombocytopenic purpura on the floor of the mouth

**Figure 2:**
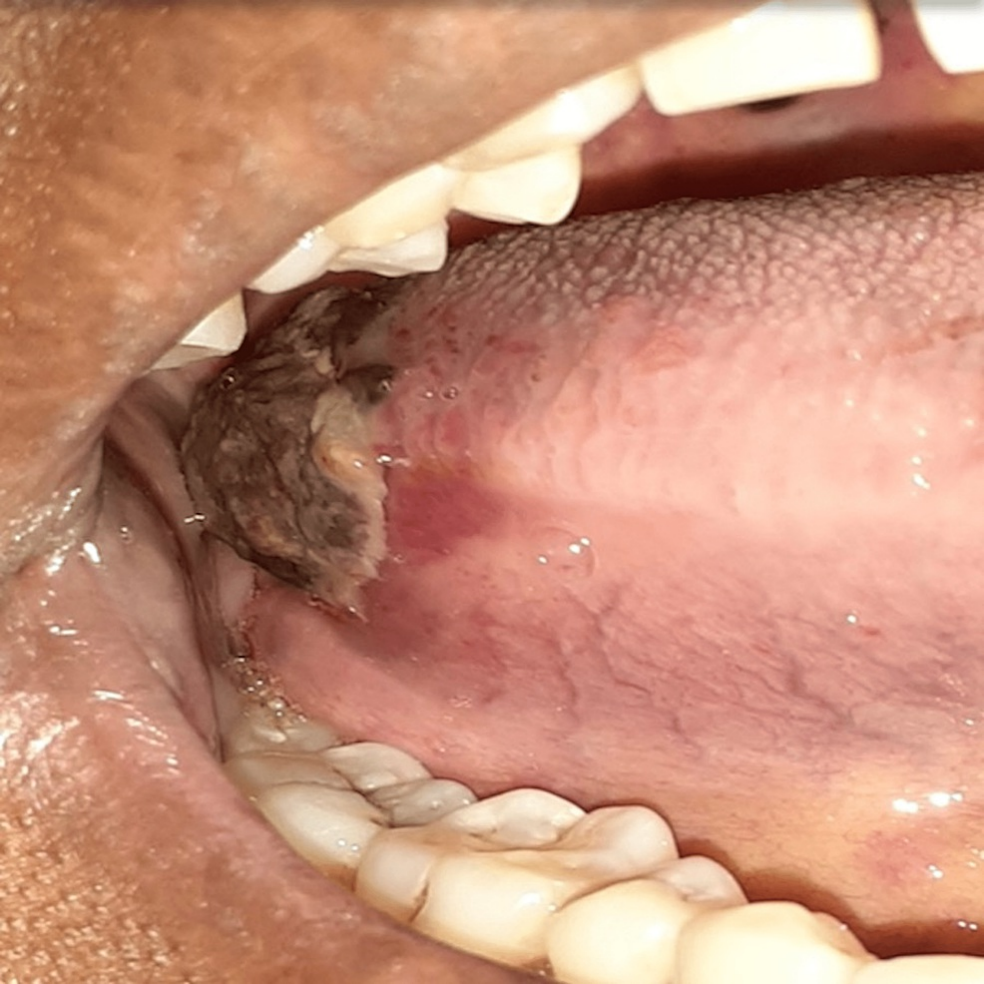
Purpura on the lateral border of the tongue

**Figure 3:**
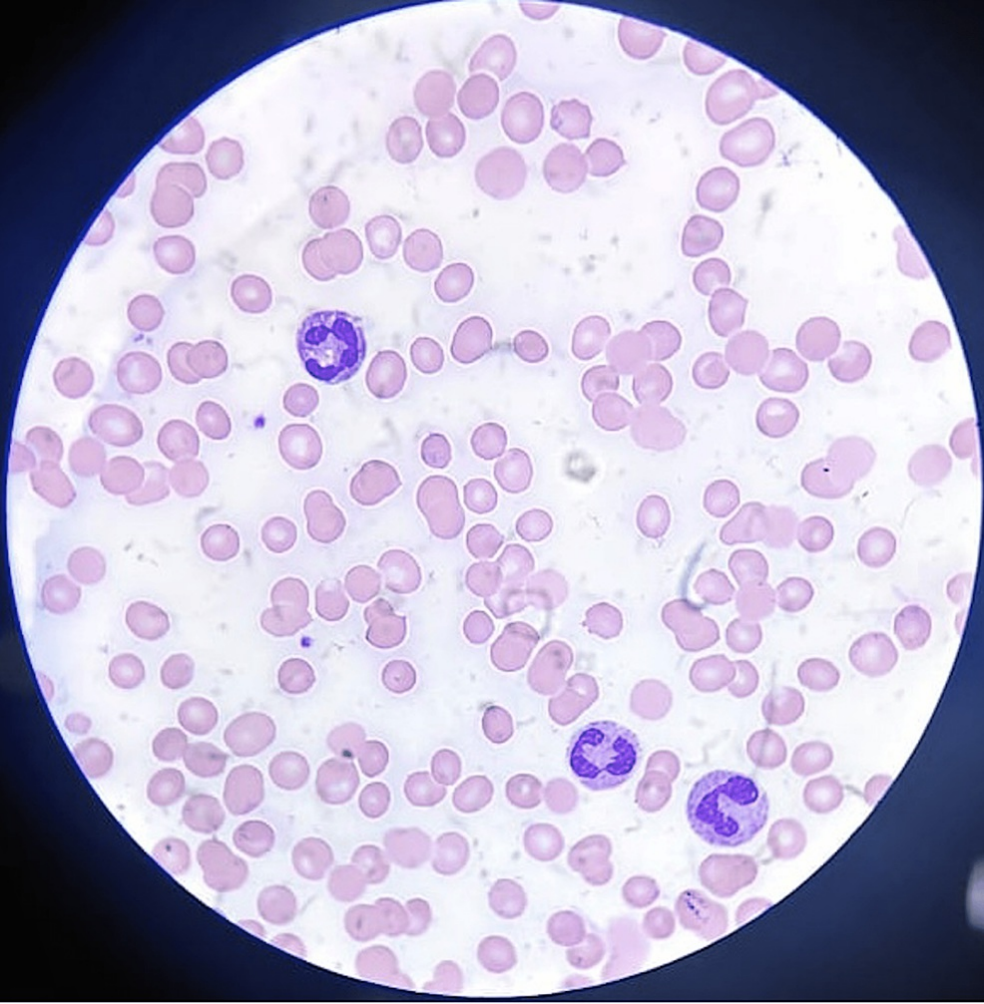
Peripheral blood smear showing thrombocytopenia

During the general examination, one cutaneous purpuric spot was observed on the ventral surface of the left upper limb. Intraoral examination revealed submucosal ecchymosis in the upper and lower lip, the anterior floor of the mouth (Figure 1), and the right lateral border of the posterior one-third of the tongue (Figure 2). There was also spontaneous bleeding from the gingival surface of the left mandibular premolar region. The peripheral blood smear showed a platelet count of 15 x 10^9^/L, which was suggestive of severe thrombocytopenia (Figure 3). RBC was normocytic and normochromic, WBC was normal, and hemoglobin level, coagulation profile, and liver and renal profile were normal. Phenytoin was immediately discontinued and replaced with sodium valproate 200 mg twice daily. Seven units of platelet transfusion were administered over a period of two days. The intraoral hemorrhage stopped on the next day and there were no new hemorrhagic episodes noted. The platelet count after seven days was 110 x10^9^/L and gradually increased to 190 x10^9^/L at the end of two weeks after discontinuing phenytoin. The patient was informed to be under regular follow-up and to report immediately if there are any new symptoms.

## Discussion

Many drugs, herbal preparations, and nutritional supplements can cause thrombocytopenia by decreasing platelet production or increasing platelet destruction, or by eliminating platelets from the peripheral blood [7]. Considering the severity of DIT, it should be considered in the differential diagnosis of a patient with thrombocytopenia who is receiving medication. Definitive diagnosis of DIT can be difficult, particularly in its immune-mediated forms. The diagnosis is still largely made by the exclusion of other etiologic factors of thrombocytopenia and by correlation of the timing of thrombocytopenia with the administration of an offending medication [8].

Though bleeding severity is inversely proportional to platelet count, episodes of bleeding are not always associated with the severity of thrombocytopenia. Patients with a platelet count less than 10 x 10^9^/L usually present with severe purpuric lesions and hemorrhagic manifestations [9].

Clinical criteria for diagnosing DIT were published by George et al. in 1998. The clinical criteria are as follows: (i) administration of the drug precedes thrombocytopenia; (ii) withdrawal of the drug results in complete recovery from thrombocytopenia; (iii) the proposed drug alone was used before the onset of thrombocytopenia, or in patients under multidrug therapy continuing or reintroducing other drugs after withdrawing the proposed drug results in sustained normal platelet count; (iv) other causative factors for thrombocytopenia were excluded; and (v) recurrence of thrombocytopenia after re-administration of the proposed drug [10].

Dr. James N George developed a web resource called “Platelets on the Web,” which lists more than 300 drugs with which at least one confirmed or suspected case has been reported to cause DIT [7]. Many reports show anticonvulsant drugs are one of the most notable etiologic factors in DIT. Phenytoin-induced thrombocytopenia was first reported in 1957 by Paoletti et al. in a 35-year-old male with a decreased platelet count of 15 x 10^9^/L two weeks after phenytoin initiation [11].

The phenytoin metabolite, epoxide, covalently bonded to the platelet surface produces antiplatelet antibodies, which lead to the destruction of platelets. This immunologic reaction induces platelet-specific drug-dependent antibody, which recognizes glycoprotein IIb-IIIa or less commonly Ib-IX membrane glycoprotein complexes. The formation of metabolites and then the binding and subsequent immune response would account for the delay in the onset of immune reactions and the onset of clinical manifestations of thrombocytopenia [6].

The mean delay in the onset of immune-mediated thrombocytopenia is reported to be one to three weeks following exposure to the drug. Few authors report the time of onset to vary from two days to two years after phenytoin initiation [6]. This case presented with a platelet count of 15 x 10^9^/L, suggestive of severe thrombocytopenia after three months of exposure to phenytoin, which improved on discontinuation of the drug therapy. Other blood investigations were normal.

Since the patient presented with severe oral manifestations with a platelet count of 15 x 10^9^/L, seven units of platelet transfusion were administered over a period of two days. The symptoms of the patient started to decline following 24 hours after the withdrawal of phenytoin and the start of platelet transfusion. Platelet count after seven days was 110 x 10^9^/L and was gradually improved to normal at the end of two weeks. No new episodes of bleeding were noted for over a month of follow-up.

According to George et al.'s clinical criteria, phenytoin is considered an etiological factor in thrombocytopenia in this patient [10]. The patient was prescribed sodium valproate for seizure prophylaxis under the guidance of a general physician. As we cannot exclude the possibility of valproate-induced thrombocytopenia, the patient was advised regular follow-ups.

## Conclusions

Phenytoin is one of the commonly prescribed drugs in seizure prophylaxis following head injuries. Phenytoin-induced thrombocytopenia is a serious and rare complication that can occur within one to three weeks following exposure to the drug. In this case, phenytoin-induced thrombocytopenia presented with multiple oral hemorrhagic manifestations and one purpuric spot on the left upper arm, without any signs of epistaxis, hematuria, melena, or menorrhagia after three months of phenytoin initiation. Oral examination for purpuric spots or gingival bleeding plays an important role in the diagnosis of the disease. As thrombocytopenia is asymptomatic unless severe, careful analysis of the clinical and laboratory findings leads to correct diagnosis and appropriate patient care. Close monitoring of blood counts may be necessary for all patients who receive phenytoin, as delay in diagnosis or withdrawal of the drug can be life-threatening.

## References

[1] Vipan WH (1865). Quinine as a cause of purpura. Lancet.

[2] Keppel Hesselink JM, Kopsky DJ (2017). Phenytoin: 80 years young, from epilepsy to breast cancer, a remarkable molecule with multiple modes of action. J Neurol.

[3] Merritt HH, Putnam TJ (1984). Sodium diphenyl hydantoinate in the treatment of convulsive disorders. JAMA.

[4] Pedersen-Bjergaard U, Andersen M, Hansen PB (1997). Drug-induced thrombocytopenia: clinical data on 309 cases and the effect of corticosteroid therapy. Eur J Clin Pharmacol.

[5] Blackburn SC, Oliart AD, García Rodríguez LA, Pérez Gutthann S (1998). Antiepileptics and blood dyscrasias: a cohort study. Pharmacotherapy.

[6] Thorning G, Raghavan K (2007). Fatal phenytoin-induced thrombocytopaenia in a neurosurgical patient. Eur J Anaesthesiol.

[7] Vayne C, Guéry EA, Rollin J, Baglo T, Petermann R, Gruel Y (2020). Pathophysiology and diagnosis of drug-induced immune thrombocytopenia. J Clin Med.

[8] Kenney B, Stack G (2009). Drug-induced thrombocytopenia. Arch Pathol Lab Med.

[9] Aster RH, Curtis BR, McFarland JG, Bougie DW (2009). Drug-induced immune thrombocytopenia: pathogenesis, diagnosis, and management. J Thromb Haemost.

[10] George JN, Raskob GE, Shah SR, Rizvi MA, Hamilton SA, Osborne S, Vondracek T (1998). Drug-induced thrombocytopenia: a systematic review of published case reports. Ann Intern Med.

[11] Brown JJ, Chun RW (1986). Phenytoin-induced thrombocytopenia. Pediatr Neurol.

